# The Efficacy and Safety of Anlotinib in Pediatric Patients With Refractory or Recurrent Solid Tumors

**DOI:** 10.3389/fphar.2022.711704

**Published:** 2022-03-31

**Authors:** Suying Lu, Ye Hong, Huimou Chen, Liuhong Wu, Feifei Sun, Juan Wang, Jia Zhu, Yi Que, Lian Zhang, Zijun Zhen, Xiaofei Sun, Junting Huang, Yizhuo Zhang

**Affiliations:** ^1^ State Key Laboratory of Oncology in South China, Collaborative Innovation Center for Cancer Medicine, Sun Yat-sen University Cancer Center, Guangzhou, China; ^2^ Department of Pediatric Oncology, Sun Yat-sen University Cancer Center, Guangzhou, China

**Keywords:** anlotinib, refractory or recurrent, pediatric, solid tumors, efficacy, safety

## Abstract

**Objective:** Refractory or recurrent pediatric solid tumors lack effective treatments, and are associated with dismal outcomes. Hence, there is an urgent need for a novel therapeutic strategy. This study aimed to evaluate the efficacy and safety of anlotinib, a novel oral multi-kinase angiogenesis inhibitor, in pediatric patients with refractory or recurrent solid tumors.

**Methods:** This single-institutional, observational retrospective study was conducted in Sun Yat-sen University Cancer Center, China. Refractory or recurrent pediatric solid tumor patients treated with anlotinib between 2018 and 2020 were evaluated.

**Results:** Forty-one and 30 patients were enrolled to evaluate the efficacy and safety of anlotinib, respectively. There was partial response in five patients, stable disease in 22 patients, no patient with complete response, with an objective response ratio of 12.2% (5/41; 95% CI 1.7-22.7). The disease control rate was 65.9% (27/41; 95% CI 50.7-81) and the median progression-free survival was 2.87 months (95% CI 0.86-4.88). The incidence rates of any grade and grade 3–4 adverse events were 80% (24/30) and 23.3% (7/30), respectively. Bleeding (20%, 6/30), hand-foot syndrome (16.7%, 5/30), and diarrhea (13.3%, 4/30) were the most common adverse events. Grade 3–4 adverse events included hypertension, hand-foot syndrome, diarrhea, anemia, and thrombocytopenia. There were no adverse events-related deaths.

**Conclusion:** For heavily pretreated pediatric solid tumors, anlotinib monotherapy and its combination with chemotherapy may be an effective treatment option with tolerable adverse events. It is necessary to monitor blood pressure when using anlotinib in children.

## Introduction

Each year, approximately 429,000 people aged 0–19 years develop cancer (Lam et al., 2019), a common cause of death in children. With the development of effective treatment strategies, the survival rate of patients with pediatric solid tumors has improved considerably (Bhakta et al., 2019). However, there are still limited treatment options for refractory or recurrent pediatric cancers, which lead to poor outcomes (Büyükkapu Bay et al., 2019). Thus, there is an unmet need for the effective and safe treatment of these patients.

It is well known that angiogenesis plays an essential role in the occurrence and development of cancers (Folkman, 1971; Carmeliet and Jain, 2011; Viallard and Larrivée, 2017; Lugano et al., 2020). Several anti-angiogenic drugs, such as bevacizumab, pazopanib, lenvatinib, sunitinib, and apatinib have been approved for many types of cancer (Geng et al., 2018; Ferrari et al., 2019; Garcia et al., 2020; Hao and Wang, 2020). Our previous study also showed that apatinib, an oral vascular endothelial growth factor receptor-2 (VEGFR-2) tyrosine kinase inhibitor (TKI), was a promising treatment option for recurrent and refractory pediatric solid tumors (Sun et al., 2020).

Anlotinib is a novel, small-molecular, oral multikinase angiogenesis inhibitor, which has a similar but broader target than apatinib. Anlotinib inhibits cancer angiogenesis and proliferation by targeting VEGFR1, VEGFR2, VEGFR3, c-kit, platelet-derived growth factor receptor (PDGFR), and fibroblast growth factor receptor (FGFR) (Lin et al., 2018; Shen et al., 2018; Xie et al., 2018; Tian et al., 2020). In 2018, it was approved by the National Medical Products Administration (NMPA) as a third-line treatment for non-small cell lung cancer (NSCLC) (Chen, 2019). Anlotinib has also shown efficacy and manageable safety in other refractory advanced solid tumors in adults, including soft-tissue sarcoma, medullary thyroid carcinoma, renal cell carcinoma, and esophageal squamous cell carcinoma (Sun et al., 2016; Chi et al., 2018). Then in 2019, anlotinib was approved for alveolar soft part sarcoma, clear cell sarcoma, and as a second-line treatment for other soft tissue sarcomas post first-line chemotherapies including anthracyclines (Gao et al., 2020). In addition, anlotinib was also approved for small cell lung cancer and medullary thyroid cancer.

To date, there is limited clinical research focusing on the use of anlotinib in pediatric patients. In this study, we aimed to evaluate the efficacy and safety of anlotinib in pediatric patients with refractory and recurrent pediatric solid tumors.

## Patients and Methods

### Eligibility Criteria

We retrospectively reviewed the clinical data of refractory or recurrent pediatric solid tumors patients treated with anlotinib in Sun Yat-sen University Cancer Center from 2018 to 2020. The inclusion criteria included: 1) less than 18 years old at the initial onset of cancers; 2) imaging or pathology confirmed progression or recurrence; 3) having at least one measurable lesion according to RECIST 1.1 (response evaluation criteria in solid tumors); and 4) failure after standard treatment or at least two lines of treatment. The exclusion criteria included: 1) less than one cycle of anlotinib; and 2) incomplete data.

The study protocol was approved by the institutional review board and ethics committee of the Sun Yat-Sen University Cancer Center.

### Treatment Schedule

Patients were classified into three subgroups according to the therapeutic regimens: anlotinib monotherapy (A), anlotinib combined with immune checkpoint inhibitor treatment (A+ ICI), and anlotinib combined with salvage combination chemotherapy (A+ SC). Oral anlotinib was given at a dose of 8 mg (<35 kg), 10 mg (≥35 kg), and 12 mg (age >18 years old) once daily, 2 weeks on/1 week off.

### Assessment of Response and Toxicity

RECIST 1.1 was used to evaluate the responses to anlotinib, while NCI CTC v5 (National Cancer Institute Common Toxicity Criteria for Adverse Events version 5.0) was used to grade the adverse events of anlotinib. Objective response ratio (ORR) referred to the proportion of complete response (CR) and partial response (PR). Disease control rate (DCR) referred to the proportion of CR, PR, and stable disease (SD). Progression-free survival (PFS) was defined as the period from the beginning of anlotinib treatment to disease progression or death, whichever occurred first. The observation time of response and toxicity was from the beginning of anlotinib treatment to the occurrence of death or end of follow-up (December 23, 2020).

### Statistical Methods

All statistical analyses were performed using SPSS (ver. 25, IBM Inc, IL). Fisher exact test was used to compare ORR and DCR among different treatment groups and cancer types. Kaplan-Meier method was used to estimate survival curves.

## Results

### Patient Characteristics

A total of 52 patients were screened. Six patients received anlotinib-based treatment as first-line treatment. Five patients received anlotinib-based treatment less than one cycle. Finally, 41 patients were enrolled in this study. These patients were evaluated for objective response and thirty patients were evaluated for safety and toxicity. The baseline characteristics are presented in Table 1. The median age of anlotinib treatment initiation was 12 (2-23) years. Most patients were male (68.3%). The histopathology types included rhabdomyosarcoma (*n* = 12), neuroblastoma (*n* = 6), Ewing’s sarcoma/PNET (*n* = 4), osteosarcoma (*n* = 3), synovial sarcoma (*n* = 3), desmoplastic small round cell tumor (*n* = 3), alveolar soft part sarcoma (*n* = 3), Wilms’ tumor (*n* = 2), paraganglioma (*n* = 1), clear cell sarcoma of kidney (*n* = 1), highly differentiated sarcoma (*n* = 1), malignant peripheral nerve sheath tumor (*n* = 1), and rhabdoid tumor (*n* = 1). Twenty patients (48.8%) had metastases at initial diagnosis.

**TABLE 1:** Baseline characteristics of the patients.

Characteristics	Total (*n* = 41), n (%)
Median age (range) years	12 (2–23)
Gender	
Female	13 (31.7%)
Male	28 (68.3%)
Metastases at initial diagnosis	
Yes	20 (48.8%)
No	21 (51.2%)
Histology	
Rhabdomyosarcoma	12 (29.3%)
Neuroblastoma	6 (14.3%)
Ewing’s sarcoma/PNET	4 (9.8%)
Osteosarcoma	3 (7.3%)
Synovial sarcoma	3 (7.3%)
Desmoplastic small round cell tumor	3 (7.3%)
Alveolar soft part sarcoma	3 (7.3%)
Wilms’ tumor	2 (4.9%)
Paraganglioma	1 (2.4%)
Clear cell sarcoma of kidney	1 (2.4%)
Highly differentiated sarcoma	1 (2.4%)
Malignant peripheral nerve sheath tumor	1 (2.4%)
Rhabdoid tumor	1 (2.4%)
Number of previous chemotherapy lines	
1	9 (22%)
2	8 (19.5%)
≥3	24 (58.5%)
Median cycles of previous chemotherapy	17 (1–34)
Median follow-up time (range) months	3.3 (2.7–3.8)

Before receiving anlotinib treatment, most patients had received different treatments, including surgery, radiotherapy, and several lines of chemotherapy. The median number of cycles of previous chemotherapy was 17 (1-34). Nine, eight, and 24 patients received anlotinib as second-line, third-line, and more than third-line treatment, respectively. The treatment schedule of anlotinib included A (*n* = 16), A+ ICI (*n* = 6), and A+ SC (*n* = 19). The ICI included three kinds of programmed cell death protein 1 (PD-1) inhibitors: keytruda, toripalimab, and camrelizuzmab. The combined chemotherapy regimens included VIT (vincristine, irinotecan, temozolomide), cyclophosphamide, vinorelbine, etoposide, ifosfamide, and others.

The median follow-up time was 3.3 (2.7–3.8) months. At the end of the follow-up, four patients were continuously treated with anlotinib treatment. The reasons for 37 patients discontinuing anlotinib treatment included disease progression (25/37), adverse events (6/37), and receiving other anti-cancer treatments (6/37).

### Efficacy and Outcomes

**FIGURE 1:**
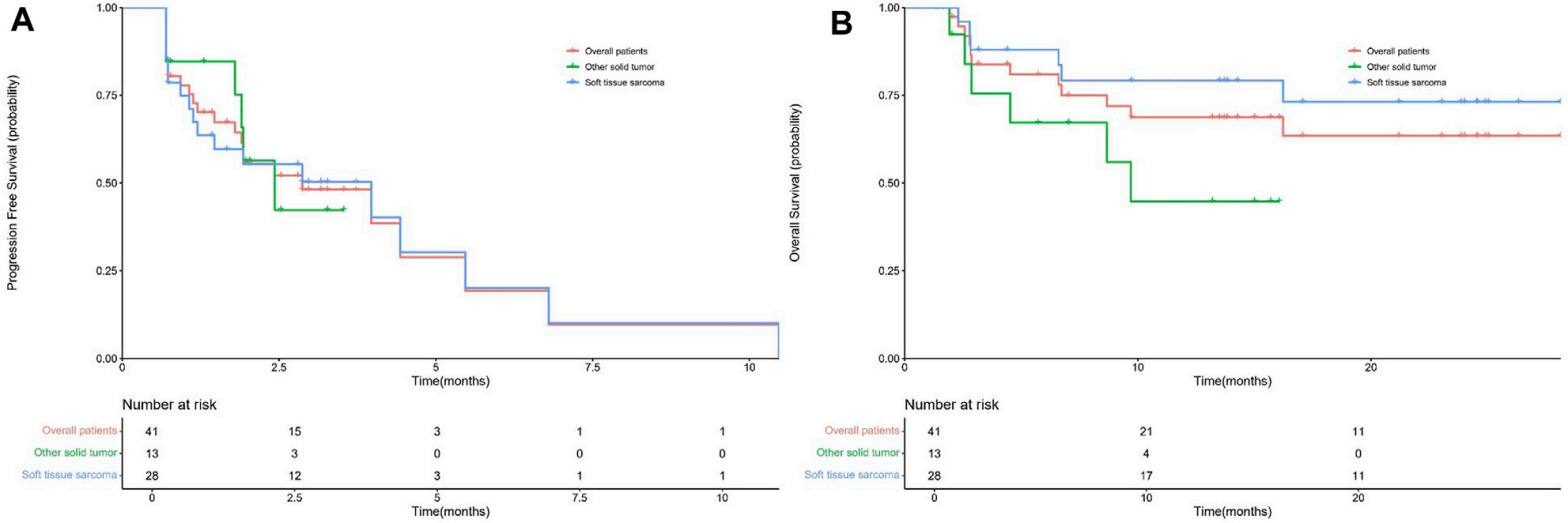
The PFS **(A)** and OS **(B)** curves for all enrolled patients, soft tissue sarcomas, and other solid tumors.

A total of 41 patients were enrolled for efficacy evaluation. None of the patients had CR. There was PR in 5 (12.25) patients, SD in 22 (53.7%) patients, and 14 (34.1%) patients developed progressive disease. As shown in Table 2, the ORR and DCR were 12.2% (95% CI 1.7-22.7) and 65.9% (95% CI 50.7-81), respectively. The median PFS was 2.87 months (95% CI 0.86-4.88) (Figure 1A). The median duration of response was 2.97 months (2.53-10.47). The maximum shrinkage percentage of the tumor was 43%.

**TABLE 2:** Treatment responses.

Clinical Evaluations	All Patients	Group a	Group A + ICI	Group A + SC
Total, n	41	16	6	19
CR, n	0	0	0	0
PR, n (%)	5 (12.2%)	1 (6.3%)	1 (16.7%)	3 (15.8%)
SD, n (%)	22 (53.7%)	8 (50%)	3 (50%)	11 (57.9%)
PD, n (%)	14 (34.1%)	7 (43.8%)	2 (33.3%)	5 (26.3%)
ORR (%, 95% CI)	12.2%, 1.7–22.7	6.3%, 7.1–19.6	16.7%, 26.2–59.5	15.8%, 2.3–33.8
DCR (%, 95% CI)	65.9%, 50.7–81	56.3%, 28.9–83.6	66.7%, 12.5–120.9	73.7%, 51.9–95.5

Abbreviations: A, anlotinib monotherapy; ICI, immune checkpoint inhibitor; SC, salvage chemotherapy; CR, complete response; PR, partial response; SD, stable disease; ORR, objective response rate; DCR, disease control rate.

The enrolled patients were divided into two groups, namely, soft tissue sarcomas (*n* = 28) and other solid tumors (*n* = 13). In the soft-tissue sarcoma group, four patients had PR and 14 patients had SD. The ORR was 14.3% (95% CI 0.5-28.1) and the DCR was 64.3% (95% CI 45.4-83.2). The median PFS was 3.97 (95% CI 0.78-7.15) months. In the other solid tumor group, one patient and eight patients had PR and SD, respectively. The ORR and DCR were 7.7% (95% CI 9.1-24.5) and 69.2% (95%CI 40.2-98.3), respectively. The median PFS was 2.43 (95% CI 1.48-3.58) months. There were no significant differences in ORR (*p* > 0.9999), DCR (*p* > 0.9999) and PFS (*p* = 0.97) between the two groups (Figure 1B).

All patients were divided into three groups according to the anlotinib treatment schedule: A (*n* = 16), A+ ICI (*n* = 6), and A+ SC (*n* = 19). The ORR, DCR and median PFS were 6.3% (1/16; 95%CI 7.1-19.6), 56.3% (9/16; 95%CI 28.9-83.6) and 2.43 months for group A; 16.7% (1/6; 95%CI 26.2-59.5), 66.7% (4/6; 95%CI 12.5-120.9) and 1.13 months for group A+ ICI; and 15.8% (3/19; 95%CI 2.3-33.8), 73.7% (14/19; 95%CI 51.9-95.5) and 2.87 months for group A+ SC, respectively. There were no significant differences in these response indices among the three groups.

### Safety and Toxicity

Due to the lack of adverse event data, only 30 patients were included in the safety and toxicity evaluation. As shown in Table 3, the incidence of adverse events was 80% (24/30). Bleeding (20%), hand-foot syndrome (16.7%), and diarrhea (13.3%) were the most common adverse events. The incidence of grade 3 or four adverse events was 23.3% (7/30), which included grade 3 hypertension (*n* = 2), grade 4 thrombocytopenia (*n* = 2), grade 3 hand-foot syndrome (*n* = 1), grade 3 diarrhea (*n* = 1), and grade 3 anemia (*n* = 1). Among the seven patients who developed grade 3–4 adverse events, one patient received A treatment (grade 3 hypertension) and the remaining six patients received A+ SC treatment. This suggests that diarrhea, anemia, and thrombocytopenia may be associated with chemotherapy. At the end of follow-up, six patients discontinued anlotinib treatment because of adverse events, including optic nerve disorder (*n* = 1), diarrhea (*n* = 1), pain (*n* = 1), hypoadrenalism (*n* = 1), hypertension (*n* = 1), and bleeding (*n* = 1). No treatment-related death was found.

**TABLE 3:** Possible treatment-related adverse events.

AEs	Any grade	Grade 1,2	Grade 3,4
Non-hematological AEs	26 (86.7%)	22 (73.3%)	4 (13.3%)
Bleeding	6 (20%)	6 (20%)	0
Hand foot syndrome	5 (16.7%)	4 (13.3%)	1 (3.3%)
Diarrhea	4 (13.3%)	3 (10%)	1 (3.3%)
Vomiting	1 (3.3%)	1 (3.3%)	0
Abdominal Pain	2 (6.6%)	2 (6.6%)	0
Seizure	1 (3.3%)	1 (3.3%)	0
Optic nerve disorder	1 (3.3%)	1 (3.3%)	0
Hypertension	2 (6.6%)	0	2 (6.6%)
Proteinuria	1 (3.3%)	1 (3.3%)	0
Skin ulcer	1 (3.3%)	1 (3.3%)	0
Adrenal insufficiency	1 (3.3%)	1 (3.3%)	0
Pain	1 (3.3%)	1 (3.3%)	0
Alopecia	1 (3.3%)	1 (3.3%)	0
Hematological AEs	4 (13.3%)	1 (3.3%)	3 (10%)
Thrombocytopenia	2 (6.6%)	0	2 (6.6%)
Anemia	2 (6.6%)	1 (3.3%)	1 (3.3%)

Abbreviations: AEs, adverse events.

## Discussion

The dismal prognosis of relapsed or refractory pediatric solid tumors highlights the importance of developing novel therapies. In this study, the efficacy and safety of anlotinib in pediatric patients with refractory and recurrent solid tumors were evaluated. The findings showed that the overall ORR and DCR were 12.2% and 65.9%, respectively.

Anti-angiogenic therapy has been recognized as an effective anti-tumor treatment in many adult tumors, including soft tissue sarcoma and lung cancer (Han et al., 2018; Chen, 2019). Anti-angiogenic therapy mainly included monoclonal antibody therapy (i.e., bevacizumab) and small molecular tyrosine kinase inhibitors (i.e., anlotinib, apatinib, pazopanib and lenvatinib). As a multiple target TKI, all the target molecules (e.g., VEGFR 1 to 3, EGFR, PDGFR-*α* and -*β* and FGFR 1–3) of anlotinib can contribute to its inhibitory action on tumor angiogenesis and affect tumor cell growth function (Lin et al., 2018; Taurin et al., 2018; Chen, 2019). A phase II study indicated that several eligible types of soft tissue sarcoma, such as fibrosarcoma, alveolar soft part sarcoma, liposarcoma and synovial sarcoma, showed high sensitivity to anlotinib [ORR = 13% (95% CI 7.6-17) and DCR = 74% (95%CI 66–8)] (Chi et al., 2018). In this study, the ORR and DCR of group A (anlotinib monotherapy) were 6.3% (95% CI 7.1-19.6) and 56.3% (95% CI 28.9-83.6), respectively. Our outcome results appeared to be worse than the above-mentioned phase II study, which might be attributed to the differences in patient characteristics between the two studies. In the above phase II study, eligible patients progressed after receiving anthracycline-based first-line chemotherapy; while most enrolled patients in our study received anlotinib treatment as fourth or more lines. Pediatric patients aged 5–18 years with nonhematopoietic solid malignant tumors, primary extracranial, that progress after at least two lines of chemotherapy were enrolled (Pramanik et al., 2017). Thirty-two patients (78%) in our study received anlotinib-based treatment after at least two lines of chemotherapy. We found that the median PFS for group A, group A + ICI, and group A + SC of these patients were 1.90; 1.13; and 2.80 months, respectively. However, the median PFS was only 46 days (95% CI, 33-58 days) in the placebo group and 49 days (95% CI, 43-59 days) in the metronomic chemotherapy group (4-drug oral metronomic regimen of thalidomide and celecoxib and with alternating periods of cyclophosphamide and etoposide) (*p* = 0.07) (Pramanik et al., 2017). Therefore, anlotinib monotherapy or combined with salvage chemotherapy may improve PFS in pediatric patients with relapsed or refractory solid tumors.

The combination of anlotinib, vincristine, and irinotecan as second-line treatment in recurrent or refractory Ewing sarcoma showed that the ORR at 12 weeks was 83.3% in 12 patients (<16 years), and was well tolerated with limited toxicity (Xu et al., 2021). Considering studies of other anti-angiogenesis treatments in childhood tumors, one study showed that the ORR for refractory and recurrent neuroblastoma patients who received bevacizumab combined with chemotherapy was only 9% (Modak et al., 2017). Compared with these studies, anlotinib achieved the best PR and SD responses in one and three out of six neuroblastoma patients in our study; and the ORR and DCR were 16.7% and 60%, respectively. This indicates that anlotinib is more effective than bevacizumab, but the findings are limited by the small sample size, which requires further investigation. Our previous study also reported that the ORR of apatinib combined with chemotherapy for refractory or recurrent pediatric solid tumor patients was 23.8% (Sun et al., 2020). In this study, the ORR was 6.3% for anlotinib monotherapy and 15.8% for anlotibnib combined with chemotherapy, suggesting that anlotibnib combined with chemotherapy may have a superior response, although it did not achieve statistical significance. Comparing the ORR of our study with previous studies of chemotherapy or anti-angiogenic treatment for refractory or recurrent solid tumors in children, the results are consistent (Sun et al., 2020). However, what cannot be ignored is that most patients received anlotinib treatment as fourth or more lines in our study, and the results of combined chemotherapy regimens are not comparable. These reasons can affect the evaluation of the response rate of anlotinib monotherapy and anlotinib combined with salvage chemotherapy. It is suggested that earlier use of anlotinib combined with chemotherapy may improve the treatment of refractory or recurrent pediatric solid tumors.

The combination of VEGF inhibitor and an immune checkpoint inhibitor (ICI) for cancer treatment has been reported. A recent study showed that anlotinib and ICI had a synergistic effect in the treatment of lung cancer (Yang et al., 2020). Another vascular inhibitor axitinib plus pembrolizumab has manageable toxicity in patients with advanced sarcomas (aged 16 years or older) particularly in those with alveolar soft-part sarcoma (ASPS), and the 3 months PFS for all patients and ASPS patients were 65.6% and 72.7%, respectively (Wilky et al., 2019). In our study, six patients received anlotinib combined with ICI. The ORR of group A + ICI was 16.7%, and the median PFS was 1.13 months, indicating that the efficacy of anlotinib combined with ICI is not optimal. The most probable reason for these results is the small sample size. Another reason is that 66.7% patients in group A + ICI received anlotinib after three lines of chemotherapy, and the median number of cycles of previous chemotherapy was 11 (1-32). Therefore, future randomized controlled trials are warranted to identify the effectiveness of anlotinib combined with ICI.

When malignant tumors progress after standard chemotherapy regimens, there are limited options available and the approach at this point involves palliative therapy, with a goal to halt the progression of cancer and improve the quality of life. A prospective study investigated metronomic chemotherapy for pediatric solid tumors that progressed after at least two lines of chemotherapy, and the results showed that the ORR and DCR were 3.5% and 17.8%, respectively (Pramanik et al., 2017). For various reasons, there are very few targeted drugs available for Chinese children with cancer. As the safety and efficacy data of anlotinib in pediatric cancers are inconclusive, anlotinib has not been approved for the treatment of pediatric cancers. So far, this is the first study of anlotinib in children using real world data. Anlotinib may be one of the few promising therapeutic options. We await the results of a phase I prospective clinical study of anlotinib, which is currently ongoing in our center. The national clinical trial (NCT) number is NCT04659733.

The general incidence rates of any grade and 3–4 grade adverse events were 80% and 23.3%, respectively, which were comparable to previous studies reported on the efficacy of anti-angiogenic therapy against tumors. Sun et al. (2020) reported that the incidence rates of any grade and 3–4 grade adverse events were 89% and 39.3%, respectively. There were no deaths related to adverse events. The most common adverse events were bleeding, hand-foot syndrome, and diarrhea. The most common 3–4 grade adverse events were myelosuppression (10%) and hypertension (6.6%), which were controlled by the use of symptomatic treatment drugs or discontinuation of anlotinib. The incidence and spectrum of adverse events of anlotinib in the treatment of refractory or recurrent tumors in children are similar to those of apatinib (Sun et al., 2020), suggesting that anlotinib treatment is safe and tolerable. However, it is necessary to pay special attention to monitoring blood pressure when using anlotinib in children.

There were some limitations to our study. First, it was a retrospective single center study and selective bias could not be ruled out. Second, the sample size was relatively small. Third, most patients received anlotinib treatment at a later point during the disease. Hence, the efficacy of anlotinib can not be thoroughly evaluated. Finally, the lack of information on adverse events precluded a detailed evaluation of these events in the study sample. Thus, a large prospective cohort study is warranted to evaluate the efficacy of anlotinib in pediatric patients with refractory or recurrent solid tumors.

## Conclusion

For heavily treated pediatric tumor patients, there was no effective treatment to choose and the main purpose was to block the growth of tumor and improve the life quality of patients. Compared with chemotherapy and other anti-angiogenic treatment options, our results showed that anlotinib monotherapy and anlotinib combined with chemotherapy may be effective treatment options with tolerable adverse events for pediatric patients with refractory or recurrent pediatric solid tumors. However, the side effects of hypertension should be alert. It appeared that anlotinib combined with immunotherapy was not beneficial to these patients. Prospective randomized clinical trials and large cohort studies are urgently warranted to verify the findings of our study.

## Data Availability

The raw data supporting the conclusion of this article will be made available by the authors, without undue reservation.
